# Categorization: The View from Animal Cognition

**DOI:** 10.3390/bs6020012

**Published:** 2016-06-15

**Authors:** J. David Smith, Alexandria C. Zakrzewski, Jennifer M. Johnson, Jeanette C. Valleau, Barbara A. Church

**Affiliations:** 1 Department of Psychology, Georgia State University, 738 Urban Life Building, 140 Decatur St., Atlanta, GA 30303, USA; jjohnson304@student.gsu.edu (J.M.J.); jvalleau1@student.gsu.edu (J.C.V.); 2 Department of Psychology, University at Buffalo, The State University of New York; Park Hall Room 204, Buffalo, NY 14260, USA; azakrzew@buffalo.edu; 3 Language Research Center, Georgia State University, 3401 Panthersville Rd, Decatur, GA 30034, USA; bchurch@gsu.edu

**Keywords:** category learning, categorization, cognitive evolution, comparative cognition, animal cognition

## Abstract

Exemplar, prototype, and rule theory have organized much of the enormous literature on categorization. From this theoretical foundation have arisen the two primary debates in the literature—the prototype-exemplar debate and the single system-multiple systems debate. We review these theories and debates. Then, we examine the contribution that animal-cognition studies have made to them. Animals have been crucial behavioral ambassadors to the literature on categorization. They reveal the roots of human categorization, the basic assumptions of vertebrates entering category tasks, the surprising weakness of exemplar memory as a category-learning strategy. They show that a unitary exemplar theory of categorization is insufficient to explain human and animal categorization. They show that a multiple-systems theoretical account—encompassing exemplars, prototypes, and rules—will be required for a complete explanation. They show the value of a fitness perspective in understanding categorization, and the value of giving categorization an evolutionary depth and phylogenetic breadth. They raise important questions about the internal similarity structure of natural kinds and categories. They demonstrate strong continuities with humans in categorization, but discontinuities, too. Categorization’s great debates are resolving themselves, and to these resolutions animals have made crucial contributions.

## 1. Introduction

Categorization is a crucial ability for humans and nonhuman animals (hereafter, animals) and a focus of research (e.g., humans [1,2,3,4,5,6,7,8]; animals [9,10,11,12,13,14]). Forming psychological equivalence classes—categories—allows consistent, adaptive behavior toward ecologically equivalent objects that have imperfect perceptual similarity. Categorization has conferred fitness advantages on vertebrates for hundreds of millions of years. For example, vervet monkeys (*Chlorocebus pygerythrus*) suffer serious mortality caused by predation—especially martial eagles (*Polemaetus bellicosus*) (e.g., [15]). Accordingly, vervets have developed call signs that warn group members to behave appropriately at the sight of eagles. In a sense, these calls denote or “name” members of the category eagle.

However, you cannot hide all the time. Vervet life must go on. So categorization needs to be discriminating and selective. Members of the category eagle must be avoided but not so vultures. Crying eagle, like crying wolf, must be rare and true so that it can maximally protect against predation while minimally disrupting foraging. So cognitive categories must be efficient: sculpted to suit the ecology, tailored to the shape of the category, sized to the extension of the category.

We discuss the nature of this sculpting within its ecological and evolutionary context. This topic has rarely been discussed. We ground our discussion in the major theories of category representation. We consider the current status of two dominant theoretical debates that arise from these contrasting theories. The debates are about how animals (and humans) might be equipped to categorize within the natural world. From this follows empirical research that illuminates how animals are equipped for categorization and *how they are not equipped*. The latter understanding is equally important. The empirical data raise important natural-history questions about categorization. They may suggest the pathways that cognitive evolution did and did not take during the emergence of cognitive systems for categorization. They may even reveal something about the structure of natural kinds that could affect animals’ fitness and help steer cognitive evolution. Throughout, we will make it plain that animals have been profoundly important ambassadors to the study of categorization. They have brought crucial theoretical insights. They have helped shape the dialog about human categorization. They have had an important role in the sea change occurring over 10 years within the categorization literature.

## 2. Three Theories

Three theories have dominated recent discussions of human and animal categorization [1,12]. They give useful structure for this chapter.

First, there is exemplar theory. This idea is that animals store the category exemplars they encounter (e.g., different specific eagles) as whole, separate memory traces. They compare new items to these, and accept them in the group if they are enough like the stored exemplars [5,7]. In this theoretical framework the organism does not have a general and unified idea of eagles. It has a slide carousel of eagles; a Rolodex of eagles. In the exemplar view, the vervet glances up, sees the thing, and then makes many psychological, similarity-based comparisons to stored memory traces (oh, it is like eagle 56; ooooh, it is very like eagle 208; and look, as it gets really close it looks amazingly like…{bad ending}). This sad allegory makes the important psychological point that one’s classification algorithm—that is, the way one’s mind is composed to process category information and behave toward it—can have serious adaptive risks. If your algorithm is too slow, too stepwise, too stringent in its acceptance criteria, the consequences can be serious. This is why there could be a premium on the evolution of efficient systems of categorization in vertebrate minds.

Second, there is prototype theory. This idea is that animals average their experiences with disparate exemplar to form the schema, the prototype, the central tendency of the category. They compare new items to this averaged cognitive representation and include them in the category if they are enough like it [16,17,18]. A prototype-based categorization algorithm also presents adaptive risks. Averaging across disparate exemplars might be difficult computationally, or take more brain, and it could be wasted brain from the perspective of exemplar theory if all one needs in memory are the individuated exemplars. Moreover, the prototype can be a poor guide to category generalization because it cannot reveal anything about the category’s boundaries or limits. These criticisms are completely fair. All theories of categorization have limitations. This could suggest that in the end organisms may turn out not to suffice with just one way of doing category business. Meanwhile, the issue is not resolved by dueling criticisms. It is a matter of empirical science. It is a question of what humans and animals do.

Third, there are category rules, grounded in an elegant cognitive-neuroscience literature [19,20,21,22]. In this area, rules are the representational vehicle for an explicit learning process with these operational characteristics. Explicit processes apprehend stimuli through focused attention to few (or one) stimulus features. They learn diagnostic features. In humans, at least, explicit processes rely on working memory and the executive functions to evaluate featural hypotheses. This learning is conscious, verbalizeable, and declarative to others. Many have granted rules an important role in human categorization [20,23,24,25,26,27]. Interestingly, even prominent exemplar theorists have done so [25,28,29].

Ashby, Maddox, and their colleagues gave explicit rule learning a neuroscience basis through their COVIS model (Competition between Verbal and Implicit Systems) of category learning [19,21,30]. COVIS models the explicit rule system as reliant on the anterior cingulate gyrus, the prefrontal cortex, the head of the caudate nucleus, and medial temporal-lobe structures also serving declarative memory. This system is modeled to resemble the neural complex that supports attention’s executive control [31]. Until recently, the phylogenetic breadth and depth of this explicit system for category rule learning remained unexplored. One sees, though, that the neuroscience scaffolding for explicit categorization just described has interesting implications for the evolutionary emergence of rule-based category learning. Not all animals have that neural complex equivalently available, and it might have emerged through evolutionary shaping during cognitive evolution.

## 3. Exemplars and Prototypes

The theories just described have provoked the two great theoretical debates in this literature. This review will take those debates in turn (Section 3, Section 4, Section 5, Section 6 and Section 7; Section 8, Section 9, Section 10, Section 11 and Section 12).

One cannot overstate the importance of the exemplar-prototype debate in categorization. Exemplar theory (*i.e.*, Rolodex theory) is an elegant theoretical possibility that plausibly could be part of the overall vertebrate system of categorization. We acknowledge our respect for this theory and for all theories within categorization.

Exemplar theory (Section 2) has exerted enormous influence over the past 30 years. For decades, exemplar theory grounded the field’s dominant theoretical narrative—that one and only one representational/processing system serves all categorization. This processing system was deemed to be exemplar-based, of course, with organisms referring new items to multiple cognitive reference points (the stored exemplars), and including them in the category if they are enough like them. This singular process had the potential to unify categorization under one mechanism. It made categorization parsimonious. It suggested that all categories are potentially learnable (even random groupings of objects could be manageable as a category through exemplar memorization). It made categorization science “easy”, with just one process in mind and memory to understand. And so exemplar theory was attractive and became powerful. Therefore, it is profoundly important that animal-cognition studies have helped resolve this theoretical debate.

## 4. Prototypes in Psychological Space

**Figure 1:**
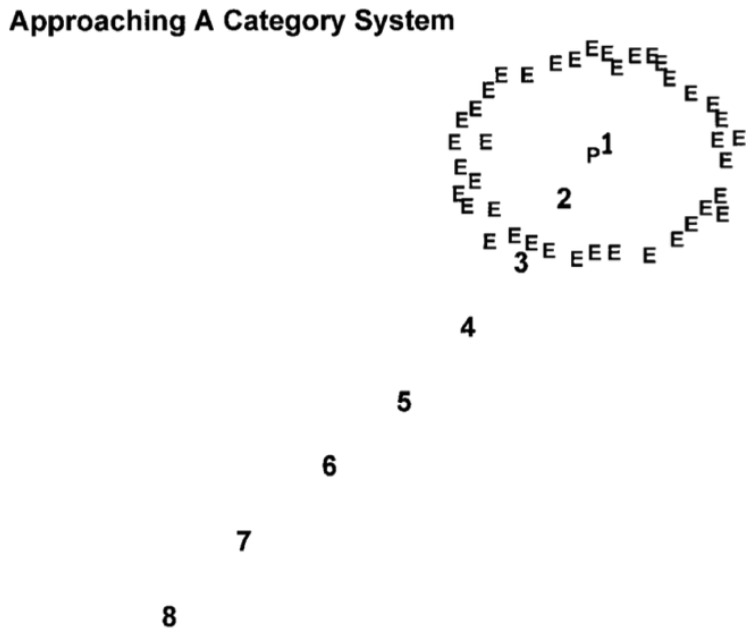
Schematic drawing of a series of hypothetical to be classified items (8 to 1) that are made to approach the psychological space of a category system containing a central prototype (P) and a shell of training exemplars (E). Reprinted from [33]. Copyright 2002 by the Association for Psychological Science. Reprinted with permission.

Suppose you are an exemplar-based vervet. You see the eagle exemplars of life. You store them in memory, spread out in the mind’s psychological space like a cloud because they are not perfectly similar (Figure 1, Es). Now you see new items that need to be classified (8…1), and, from the outside working in, you will say, impossible, no way, no, maybe, and so forth, with your potential for category endorsement increasing approaching the cloud. Just as Rosch [32] envisioned, the category would have a typicality gradient. However, this gradient has a theoretically crucial asymptote. A new item cannot ever be close to all the Es at once. It will always be near some but far from others. Even the central item 1 will still not be close to all the Es. So there might be a cap on the exemplar likeness and category belongingness of the prototype, though the prototype—as defenders of exemplar theory have noted—would be the item type most strongly classified into the category.

In contrast, suppose you are a prototype-based vervet. Now you average the Es into the central prototype P (Figure 1). The prototype is a point in the mind’s psychological space. A new item can be indefinitely close to it. There is no cap on prototype likeness. The typicality gradient need not flatten. This vervet could experience a DEFCON 1 eagle that is essentially identical to its unitary category representation.

Thus, inevitable geometrical facts about the geometry of exemplar-based and prototype-centered representations force differential predictions that let one finally resolve the exemplar-prototype question in an important empirical case.

**Figure 2:**
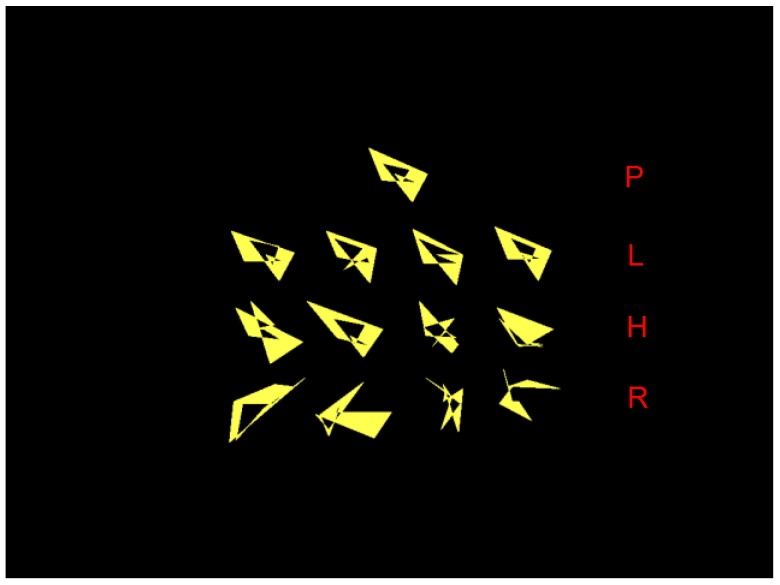
A dot-distortion category, with the originating prototype (P), low-level distortions (L), high-level distortions (H), and random-unrelated shapes (R) in the top to bottom rows, respectively. Adapted from [18]. Copyright 2008 by the American Psychological Association. Reprinted with permission.

To show this, Smith *et al.* asked macaques to learn about families of shapes [18]. These shapes were created using the influential dot-distortion or random polygon technique of Posner, Homa, and others [17,34]. These shape families were built from a prototype P (Figure 2). There could be low-level distortions of the prototype, quite similar to it, high-level distortions, less similar, and random items completely dissimilar from the prototype that should not be included within the category. The monkeys successively learned 10 shape categories in each of two experiments.

**Figure 3:**
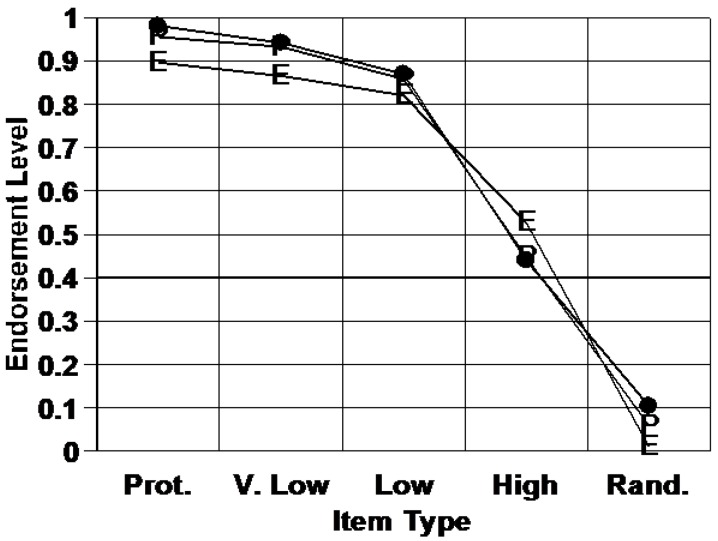
The proportion of times a macaque endorsed into a learned category to-be-categorized items that were outside the category (Rand.), non-typical category members (High-level distortions), typical members (Low-level distortions), highly typical members (V. Low-level distortions), or prototypical members (Prot.). Standard exemplar and prototype categorization models fit the macaque’s performance as well as they could (E symbols and P symbols, respectively). The models differ only in their assumption of multiple, specific-exemplar cognitive reference points for categorization, or a single, central cognitive reference point for categorization. From [18]. Copyright 2008 by the American Psychological Association. Reprinted with permission.

The filled symbols in Figure 3 show how often monkeys made category-endorsement responses for polygon shapes at different distortion levels from the prototype—that is, how often they included those shapes within the shape family or category they had learned. The performance on prototype items was essentially as high as it could be. There seems to have been no cap on category endorsement for the prototype as exemplar theory might predict.

More telling, the E symbols show how an exemplar-based macaque should have performed in this task. The exemplar model is capped too flat. It’s off by 10% much of the time. In this literature, this model fit is quite poor. Moreover, Smith *et al.* conducted additional simulations and model-fitting studies to try to improve the exemplar model’s fit [18]. They failed, because the exemplar model is up against the inherent geometry of multiple exemplars spread out in psychological space. Macaques simply are not exemplar-based in this task.

The P symbols show how a prototype-based macaque should perform. This model was like the exemplar-based model just described, except now the psychological assumption was made that macaques were comparing items needing categorizing to a unitary category representation that was the average of the training exemplars. The prototype model fits extremely closely. There is a strong possibility that macaques are prototype-based in this dot-distortion task, though this does not mean they always are. For mathematical reasons, the dot-distortion task provides a particularly strong contrast between prototype and exemplar processing. However, we point out that the same conclusion has been reached in numerous earlier animal-cognition studies [35,36,37,38,39].

## 5. Prototypes: The Trouble with Exceptions

**Figure 4:**
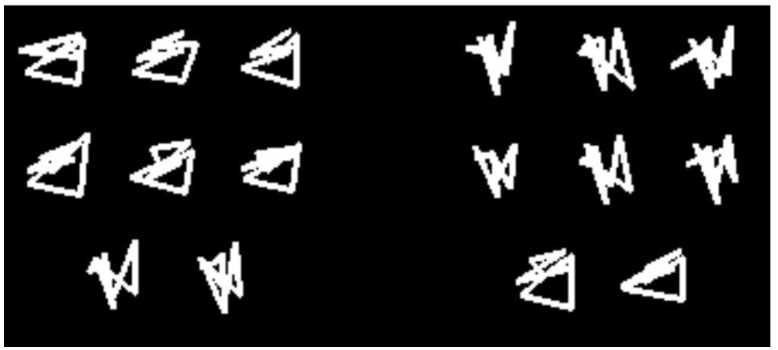
Examples of categories and stimuli used in Smith *et al.* [40]. The eight-shape groupings are two categories A and B. The six shapes in the top rows of each category are variations on the theme of the category prototype. The bottom row contains exception items that are variations on the theme of the opposing prototype. From [40]. Copyright 2010 by the American Psychological Association. Reprinted with permission.

A prototype-exception task (Figure 4) also lets one explore animals’ commitment to prototypes. Here, the shapes on the left are Category As, though the last two do not appear to be. These items are the exceptions. The same holds for the Category B items to the right.

If animals just store separate exemplars, they will potentially be OK in this task. They will be able to memorize all the Category A and Category B exemplars. They will learn to respond correctly to typical items (those clearly in the category) and exception items (those questionably in the category).

However, they will not if they take a prototype-based approach to categorization. Then they will call the Category A exceptions Bs, as their appearance suggests (and similarly with the Category B exceptions). If we observe poor performance with criss-cross exceptions like this, we will know that animals wrongly assumed two prototype-averageable groupings.

**Figure 5:**
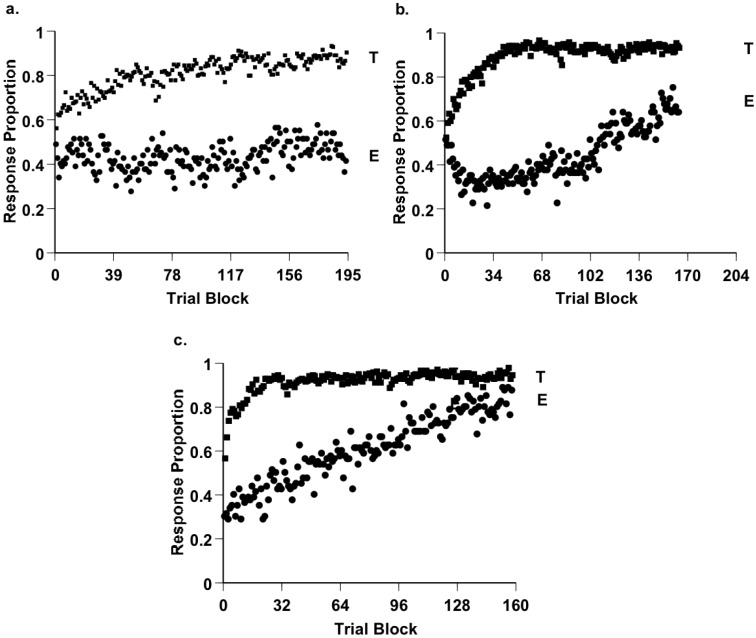
(a–c) The performance of monkeys Hank, Lou, and Murph by 64-trial blocks in the category task of Smith *et al.* [40]. Curves T and E, respectively, show the proportion of correct responses made to the six typical and two exception items in each category. From [40]. Copyright 2010 by the American Psychological Association. Reprinted with permission.

Figure 5a shows a macaque’s result over 60,000 trials in this task [40]. He was relentlessly awful on exceptions because he criss-crossed them. He did not learn them though he had 15,000 tries at them. In addition, he received 8000 correction trials on these items, because we repeated these stimuli to make sure he received the experience of making the correct response on them. Still, he did not learn though there were only four exceptions. He showed very poor ability to remember and respond to particular exemplars. If this monkey had to face exceptional eagles in his world, he would make a fatal mistake. Figure 5 gives the results from three macaques in this study. They all showed poor exception performance for thousands of trials. They seem to expect a prototype to work adaptively. They seem to default toward prototype averaging. And so they criss-cross the exceptions, because they think all stimuli should perceptually fit their prototypes. We think humans are like this, too. Humans often flail with poorly-structured, weird tasks that depend on exemplar processing. Murphy, Blair, and others have also discussed these weaknesses in our classification [41,42,43]. If you thought to yourself earlier, oh come on, this exceptions task is silly and unfair to the animals, that is you agreeing with the macaques.

If animals make a strong prototype assumption in the prototype-exception task, it is a theoretical blow to exemplar theory. And so here, that theory’s adherents should and can make a rejoinder. That is, two of three macaques learned the exceptions by the end—if you train doggedly enough, exemplar processing does finally shine through.

We agree, though we add two observations. First, we believe that the animal’s assumption as it starts a task is a crucial fact about its mind, no matter what it thinks after thousands of trials. The assumption says what the animal assumes about how the world is. In this case, animals clearly made an initial prototype assumption. Second, the late trials that show exception-item mastery unfortunately have no ecological or evolutionary relevance. Natural selection will never grant a monkey 5000 eagle-classification errors on the way to eagle mastery. In nature, your initial approach will be determinative and decisive, and on failure you do not reach the later phases. Researchers like to call a task’s final trials terminal performance, but nature takes a different view. Really, the early trials are terminal performance in its grimmer sense, if animals persist in criss-crossing exceptions, as they clearly do.

Thus, animal participants teach a very important lesson to the human categorization literature. Often it will be humans’ initial, intuitive approaches to category tasks that reveal the psychological essence of their category-learning system. Often it will be less illuminating to make them perform thousands of difficult, unnatural trials. Even if they learn it, we have then studied categorization at its unnatural extreme.

**Figure 6:**
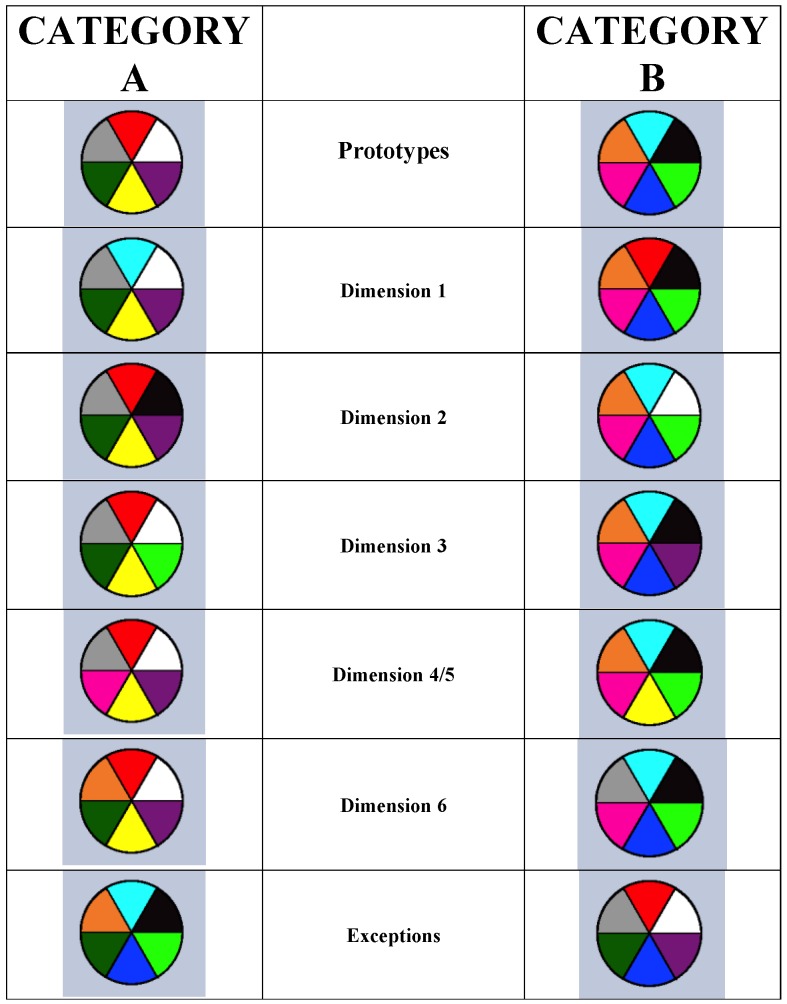
Illustrating the prototype-exception task used to test pigeons and humans in Cook and Smith [44]. Each category contained a prototype (Row 1), five typical stimuli (Rows 2–6—five features shared with their prototype), and an exception (Row 7—five features shared with the opposing prototype). From [44]. Copyright 2006 by the Association for Psychological Science. Reprinted with permission.

Cook and Smith showed that the prototype assumption may extend widely across the vertebrates [44]. In their prototype-exception task (Figure 6), the prototypes had six typical colors. The typical items in Rows 2–6 had five. The exception items in Row 7 had just one, producing the intuition that they should belong to the other category. When we say “should,” now we are agreeing with the macaques in Smith *et al.* [40].

**Figure 7:**
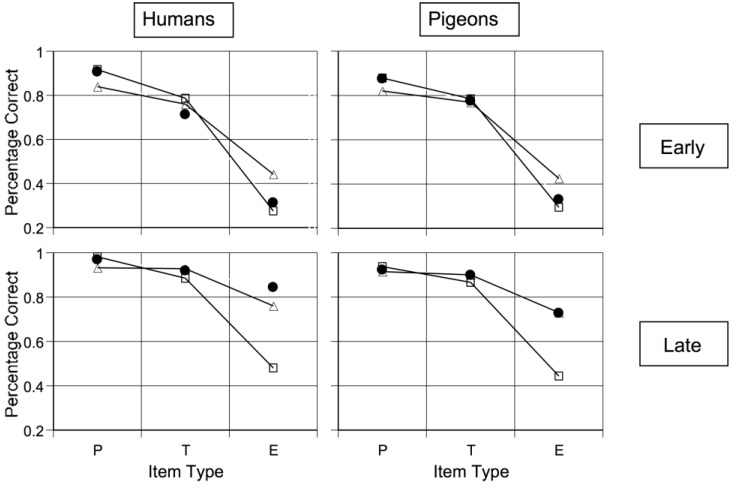
Humans’ and pigeons’ observed and predicted accuracy for prototypes, typical items, and exceptions during the early *(top* panels) and late (*bottom* panels) stages of learning within the prototype-exception task of Cook and Smith [44]. Observed performances are shown as unconnected, filled, black circles. The best-fitting predictions of prototype and exemplar models are shown, respectively, as connected open squares and open triangles. From [44]. Copyright 2006 by the Association for Psychological Science. Reprinted with permission.

The black dots in Figure 7 show early learning in this task. Early on, humans and pigeons criss-crossed the exceptions, as macaques do. Cook and Smith also fit formal models to these data [44]. The triangle symbols show what an exemplar-based categorizer should have done in this task. The typicality gradient across exception, typical, and prototypical items is flattened too much. Exemplar processing is not what humans and pigeons were using at this stage. The square symbols show what prototype processing categorizers should have done. This model fit well. Possibly both species made some kind of a prototype-based assumption at this stage. Thus, something like a prototype or abstractionist assumption spans many vertebrate evolutionary lines, giving that assumption evolutionary depth. Wasserman, Kiedinger, and Bhatt found an important converging result that they grounded in especially intuitive and elegant psychological models [14].

Cook and Smith [44] also found that, ultimately, the character of processing changed for both species. Now exemplar memorization finally became successful, exception-item performance improved, the exemplar model fit better, and the abstraction model progressively failed. Smith *et al.*’s macaques showed this same pattern, suggesting a residual capacity to finally learn exceptions is also common in vertebrates [40].

## 6. Why Prototypes

Now we should ask why vertebrate species might make a default prototype-based assumption entering category tasks. To this end, Smith asked how well exemplar and prototype minds would function in the real, ecological world [8]. He considered how well these processes might work for avoiding eagles, which would be the point of having an eagle category and communicating about it.

To ask this question was a somewhat radical step in this field. For decades, the literature had the dominant narrative that all categorization is exemplar based. Given that narrative, there would not be different processes in different minds that are differently effective. These possibilities have no place in a unified field theory of exemplar categorization. It is an important lesson, too, that dominant narratives of this kind can have a suppressing, censoring effect.

**Figure 8:**
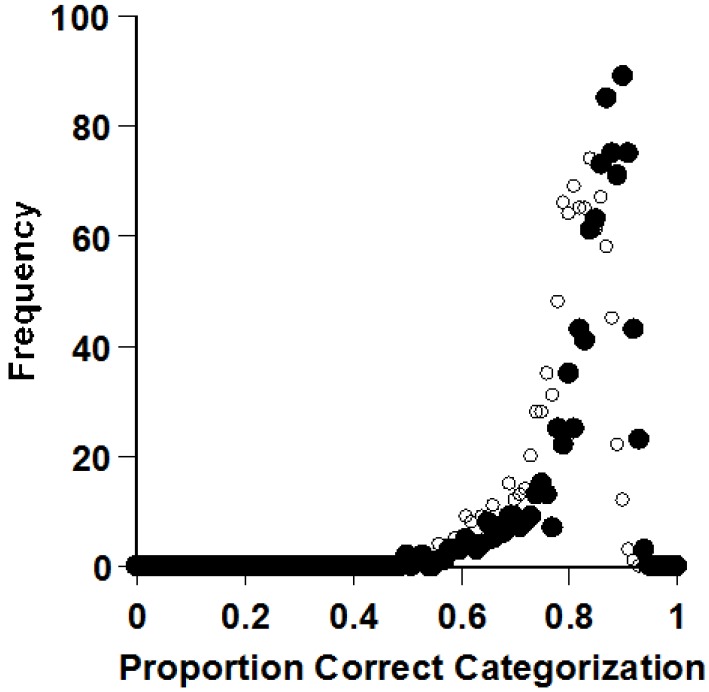
The frequency with which the prototype and exemplar process (filled and open circles, respectively) categorized transfer items at different accuracy levels in a family-resemblance task. Reprinted from [8]. Copyright 2014 by the Psychonomic Society. Reprinted with permission.

Smith did extensive simulations [8]. He created thousands of virtual exemplar and prototype monkeys, with the idea of evaluating who categorizes most efficiently. Figure 8 shows one result of many in a series of extensive simulations. Prototype processers—the black dots—averaged 84% correct categorization in Smith’s simulations. Exemplar processors—the clear dots—averaged 80%. These means were far apart statistically. As Smith varied his simulated tasks in many ways, prototypes almost always provided the most efficient strategy. Smith even showed that the prototype effect emerges instantaneously, as soon as multiple exemplars have been experienced. That is, even using the average of just two exemplars as a standard in categorization is better than using two exemplars separately as standards. Prototypes very often—not always!—give the clearest signals for categorization.

Why do prototypes provide the best signals for categorizing? The prototype is closest to all the exemplars. If you know it in your mind, you know a thing that will help you classify a lot of category members, because the prototype, in a sense, is the least-squares solution to the category problem. However, if you store exemplars, they can be distant from one another, far across psychological space, and they may not support accurate categorization as well. On average, prototypes are often closer (similar) to exemplars than exemplars are closer (similar) to exemplars. Another point is that exemplars contain both category-level features and exemplar-idiosyncratic features, so that exemplars are part category signal and part perceptual noise. The noise can deceive, distract, and mislead (though in rare circumstances, when the next exemplar is much like the idiosyncratic last exemplar, this may support accurate categorization). In contrast, prototypes that are well constructed have no idiosyncrasies, but only category essence. They are pure signal and they will not deceive. The advantage they confer is not rare and specific, either, but frequent and general across the psychological space of the category.

With these considerations in mind, reflect on vertebrate evolution over 500 million years. Prototypes might have given organisms a relentless small edge in recognizing ecologically important things. It could have meant more food, fewer eagles, better survival, more fitness. Differential fitness could have shaped our categorization processes by selecting for those with better prototype categorization skills. This would explain the prototype effects that many in the categorization literature have observed. This could be an illustration of Shepard’s idea that cognitive-processing systems—like physical bodies—are sculpted by the structure of the natural world [45,46].

## 7. Forcing the Exemplar Assumption

Perhaps the artificial prototype-exception categories in Smith *et al.* and Cook and Smith were inherently deceptive to animals, causing them to adopt a prototype-based approach because it seemed intuitively sufficient though it was not [40,44]. Another approach toward addressing animals’ categorization strengths and weaknesses is to force them to make the exemplar assumption that one wants to evaluate, not the assumption they might spontaneously prefer.

**Figure 9:**
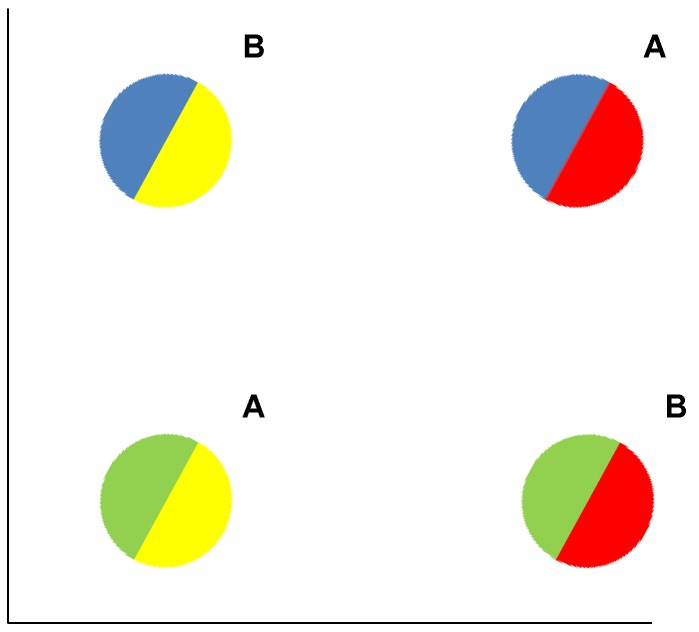
An exclusive-or (XOR) category task. Category A members have both red and blue sectors or neither and both yellow and green sectors or neither. Category B members have both blue and yellow sectors or neither and both red and green sectors or neither. Single sectors do not predict category membership and categories do not share family resemblance.

The exclusive-or (XOR) task suits this purpose (Figure 9). In this task, prototype averaging will completely fail. The “average” of the A and B items will be identical, yielding no distinguishing category information. In this task, exemplar memorization is extremely useful. Really, all the items are exceptional, the task is perceptually chaotic. One simply has to learn the four stimuli and what responses they deserve.

**Figure 10:**
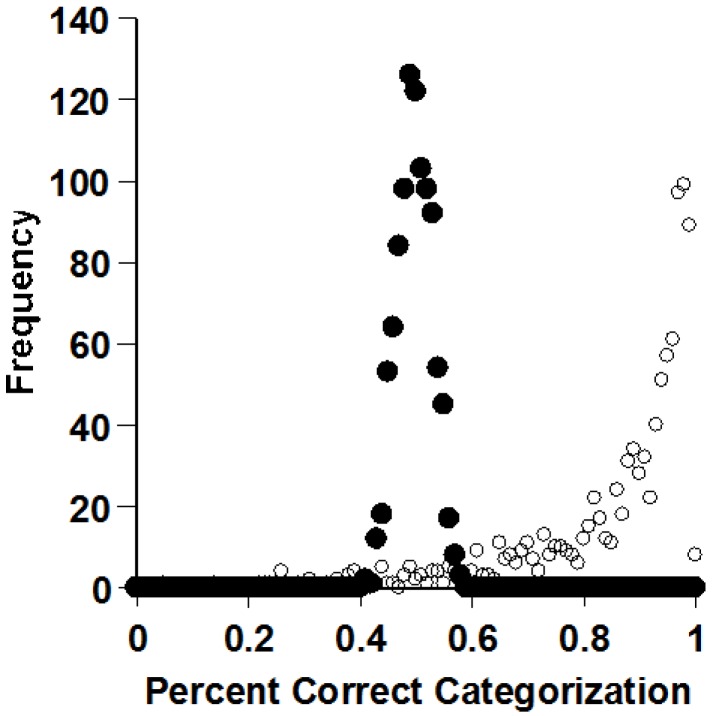
Frequency with which the prototype and exemplar process (filled and open circles, respectively) categorized transfer items at different accuracy levels in an exclusive-or category task. Reprinted from [8]. Copyright 2014 by the Psychonomic Society. Reprinted with permission.

Smith confirmed these well-known facts through simulations (Figure 10) [8]. In this task, prototype processing (black dots) is useless. It has to be. Exemplar processing (clear dots) is highly effective. So there could be times when storing exemplars confers a vital performance advantage. The XOR task is a particularly strong test of exemplar processing in categorization, because it gives animals no way to fall back to some prototype-based assumption.

Accordingly, Smith, Coutinho, and Couchman gave thousands of XOR trials to macaques [47]. They found these tasks very difficult. They were only 75% accurate by the end of training. They missed about 33% of all trials. During the experiment, they received 15,000 20-s timeouts. Remember, each error could have been a diving member of an XOR eagle category, and the consequences of an error far more serious than a timeout. Once again, macaques showed a poor capacity to store and respond differentially to individual exemplars.

**Figure 11:**
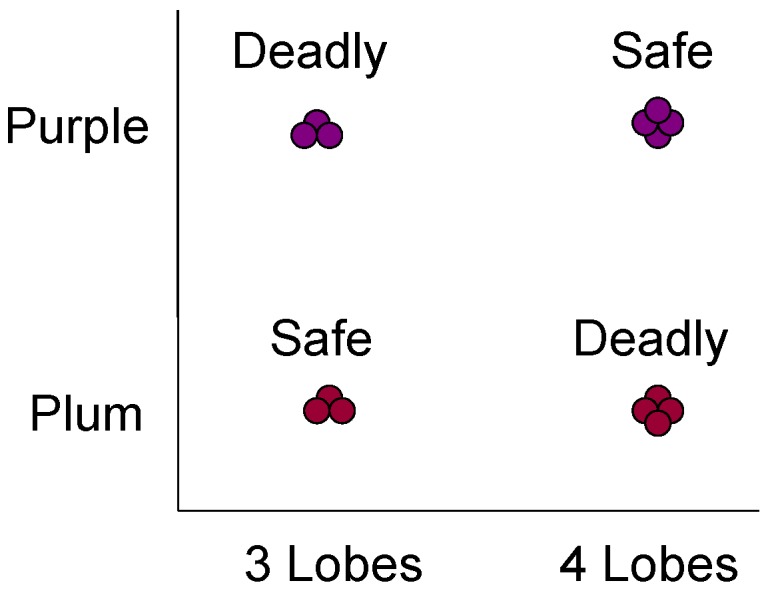
A hypothetical ecological exclusive-or (XOR) category task. To our knowledge, nature almost never presents this kind of foraging or predator-avoidance problem to living organisms, and it is a graceful state of nature that this is so.

This XOR failure has important implications. The world might be full of exceptions, XORness, and other poorly-structured categories that defeat prototype processing and demand exemplar memory—for example, the XOR berry task shown in Figure 11. How long would a foraging macaque last being only 75% correct on this task? This would be an adaptive disaster. If the world were exemplar-demanding like that, animals likely would have evolved toward exemplar adeptness, unless exemplar processing is computationally too difficult (which all exemplar theorists would reject). That they are not exemplar adept suggests the world is mainly not exemplar demanding like that (though certainly the world may sometimes be exemplar demanding, and animals sometimes exemplar processors, as when they manage the individuals within the dominance hierarchy of their group).

Instead, suppose the world is mostly prototype-averageable. Then prototype processing might be selected for and achieved during cognitive evolution, unless it is computationally too difficult (which the data disconfirm). Animals would be sensitive to prototypes. They would make default, family-resemblance assumptions. They do. They could safely be poor exemplar processors. They are. So might their human cousins be. They are. This idea explains far more of categorization’s natural history.

This idea also sharply delimits the explanatory role of exemplar theory, but its adherents have a rejoinder. The universe of logically-possible categories contains mainly poorly-structured categories. In its overall Venn diagram, prototype categories are just a sliver. So then, how could it possibly be that animals are especially adept at this tiny sliver of the possible tasks they might have to confront? Is that not an adaptive disaster and a lousy way to equip an organism for life in the natural world?

No, because the logical universe is not relevant to evolution or behavior. Only the natural world is relevant. In the natural world, it may well be that prototype categories are dominant, that animals are adept at what they need, that their prototype adeptness is not a coincidence and not an evolutionary disability. In fact, the natural kinds of the monkey ecology seem to bear this out. Their world does not give them chaotic XOR berry tasks. It gives eagles, leopards, snakes, Masai, insects, mates, and so forth—all coherent and possibly prototype-averageable things. Drawing on many psychological and anthropological studies, Malt provided a seminal review of the structure that may be given to humans and animals through natural kinds [48].

This discussion suggests that there has been an evolutionary tuning between prototypes in mind and nature. Animals have made us look at what is naturally true, what performances are naturally optimal, in ways the literature had not considered. In this area, understanding categorization’s natural history is crucial, and animals have been great behavioral ambassadors for bringing these ecological issues to the fore. Indeed, animals could even be saying tacitly that really vertebrates haven’t been doing much exemplar processing for hundreds of millions of years. This is a literature-changing insight. In the end, a unitary exemplar account of categorization simply cannot stand.

Nonetheless, exemplar theory could have some role within vertebrates’ overall categorization capability. There are times—as in managing dominance relations (for monkeys in a troop, for humans in a modern workplace)—when individuals must be encoded individually. There are sparse categories (e.g., the category of Crosby, Stills, Nash, and Young songs) whose members are encoded individually. We have often stated explicitly that the structure of laboratory category tasks can also move participants toward the pole of prototype abstraction or toward the pole of exemplar memorization [49]. It might be a useful rule of thumb for readers to see that category representations are memories. Different category-learning systems might map onto different memory systems (e.g., exemplars onto episodic memories). This is why the systems debate considered now looms so important within our field.

## 8. Category Rules and the Single-System, Multiple System Debate

Now, we turn to the evolutionary history of the third major representational system that may support category learning—category rules. Cognitive scientists and neuroscientists have considered whether humans and animals approach categories with an ability to selectively attend, find a single predictive cue of category membership, form a cognitive rule based on that diagnostic feature, and use it to classify systematically and economically. The idea of a rule-based system for categorization has been deeply controversial in the categorization literature (e.g., [50,51,52]), and it has provoked a lasting debate about single *vs.* multiple systems of category learning (*i.e.*, a single exemplar system *vs.* multiple systems comprising exemplars, rules, and prototypes). Clearly, category rules are completely different from stored exemplars as category representations. Rules are cross-exemplar summaries. Rules can exist absent any exemplar storage and applied absent any exemplar comparisons. So category rules challenge the narrative of a unitary exemplar-based theory as clearly as prototypes do. Moreover, note that rules are abstractions that in some species could even go toward logical propositions, symbolic functioning, and the verbal declaration of category rules. Consequently, there is a lot of theoretical food for thought in the possibility that rules might be a sub-system within animals’ and humans’ overall system for category learning.

Considering the prototype-exemplar debate evolutionarily, animal-cognition studies revealed continuity across vertebrate lines. However, considering the rules-exemplar debate we will find, instead, strong evolutionary divergence. These stages may suggest the original character of vertebrates’ category learning. They make us reckon with the psychological meaning of constructs like “rule” and “selective attention.” They suggest the nonhuman primates as a transitional group standing in evolution near the horizon of true category rules. By documenting this evolutionary progression, animals have brought important theoretical insights to this area as well.

## 9. Rule-Based Categorization—Theoretical Context

The category-rule literature draws on a distinction between implicit and explicit processes. The implicit process apprehends stimuli holistically with diffuse attention that encompasses multiple stimulus features. It learns by associating responses to whole stimuli. These associations are behavioral-implicit. They are neither conscious nor verbalizeable. The COVIS model of Ashby and colleagues modeled the implicit process as reliant on associative-learning mechanisms. As described in Section 2, the explicit process emphasizes attention to single stimulus aspects. In humans, it relies on working memory and the executive functions to evaluate hypotheses. It learns through conscious hypothesis testing and its contents are verbalizeable. However, verbalizability may not be a requirement for explicit categorization, as will be discussed below in the context of animal-cognition research. Ashby and his colleagues have clearly acknowledged this.

Though ours is not a neuropsychological review, we point out that process distinctions like that between implicit and explicit categorization are grounded in the study of cognitive neuroscience (review in [22]). According to one neuroscience framework, humans’ explicit/analytic processes are mediated in part by a broad neural network that includes the anterior cingulate gyrus, prefrontal cortex, the head of the caudate nucleus, and medial temporal lobe structures that serve declarative memory. This brain system is related to a broader neural complex serving the executive control of attention [31]. The implicit/nonanalytic processes depend heavily on the striatum and are catalyzed by the reinforcement-mediated strengthening of dopamine-related synapses [19,20].

One can consider exemplar and prototype processing from the same neuroscience perspective. It is known that in humans’ processing in category tasks changes as one increases the number and density of exemplars in categories. For sparse categories, something more like exemplar memorization prevails. For densely populated categories, something more like prototype abstraction prevails [17,41,53]. Antzoulatos and Miller [54] explored the neuroscience of this transition in monkeys. They found that early on, when the monkeys could acquire more specific stimulus-response associations, or one could say exemplar-response associations, striatum was the earlier predictor of category responding. Later on, as the number of exemplars increased and the task became more a general task of categorization, activity in the pre-frontal cortex began to predict category responding earlier. This result parallels the idea that implicit categorization represents the linking of responses to whole stimuli—or one might say whole exemplars—and it is also more dependent on the striatum.

In our view, categorization science is strengthened greatly by the study of its neuroscience basis and by the application of systems neuroscience to dissociating its different processes and sub-systems. Clearly, this application of neuroscience is pointing toward the presence in human and primate brains of multiple, dissociable processes within the overall category-learning system.

## 10. Rule-Based Categorization—Methodology

**Figure 12:**
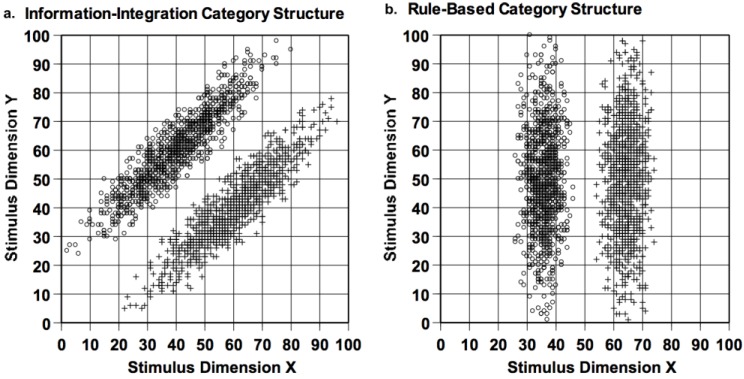
(**a**) An information-integration category structure, depicted within an abstract 100 × 100 stimulus space. The open-circle and plus-sign symbols, respectively, indicate Category A and Category B stimuli; and (**b**) a rule-based category structure, depicted in the same way.

The implicit and explicit category learning systems have been differentiated using rule-based (RB) and information-integration (II) category tasks, as depicted in Figure 12. Given the vertical category boundary in the right panel, *X*-axis variation—only—distinguishes the two categories. The participant must discover the dimensional rule (*X* < 50 (Category A); *X* > 50 (Category B)). Of course this whole stimulus space is not shown. Rather, the rule must be discovered based on feedback from successive trials. The informative dimension should be attended sharply; the non-informative dimension ignored. The left panel shows an II categorization task. Given this major-diagonal category boundary, both dimensions are partially informative. Subjects must integrate the information offered by both to produce a correct categorization response (again based on feedback from successive trials). Neither dimension can be ignored.

RB and II tasks are matched for important aspects of category structure, including between-category exemplar separation and within-category exemplar cohesion. Consequently, they are also matched for the *a priori* perceptual difficulty of the categorization problem. In fact, the two category structures are essentially identical, except for a 45-degree rotation through stimulus space. If the X and Y dimensions were truly integral (e.g., the color attributes of brightness and saturation, in which attentional processes cannot selectively target one dimension [55]), the tasks would be identical for learners, because there would be no privileged dimensional axes and neither category task would be rule-based. Thus, for many reasons, the RB and II tasks serve as strong mutual controls. They differ only in the potential value of selective attention and rule formation in one task but not the other.

In fact, this is why the RB-II dissociation can make a comparative contribution. By rotating the dimensional axis of category tasks, from II to RB, one can ask whether the cognitive systems of different species are dimensionally aligned or polarized. If they are, then the dimensional RB task may support robust and rapid learning, just as a polarizing filter will strongly admit light when aligned to the light’s polarization axis. If the cognitive system of a species is not dimensionally polarized, both II and RB tasks will be learned to the same level at the same speed—because the task’s dimensions will be integral to the species in a sense. This evaluation can be made regarding any species that can participate in operant-discrimination tasks, making the RB-II framework a valuable comparative assay.

**Figure 13:**
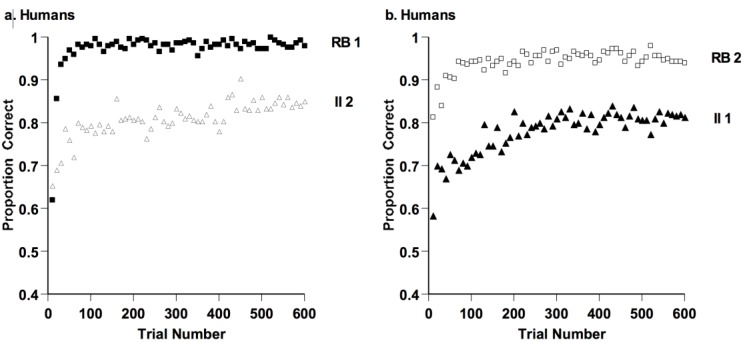
(**a**) Proportion of correct responses in each 10-trial block for 30 humans who performed 600 trials of a rule-based (RB) and an information-integration (II) category task, in that order; (**b**) Proportion of correct responses in each 10-trial block for 30 humans who performed 600 trials of an II and RB category task, in that order. Reprinted from [58]. Copyright 2012 by Elsevier. Reprinted with permission.

For example, Figure 13 shows the result when Smith *et al.* gave humans RB and II tasks [56]. The circle stimuli varied in the width and tilt of internal bars. Participants were told that they should decide whether each striped circle belonged to Category A or B. They received the RB and II tasks in counterbalanced order (RB-II or II-RB). Humans clearly showed the crucial results of learning the RB task with distinctive ease and sensitivity and producing a strong RB performance advantage. Extensive research by Ashby, Maddox, and their colleagues have produced this result many times (e.g., [57]). Moreover, Maddox, and Ashby have described many related implicit-explicit dissociative phenomena in humans’ category learning [21]. Humans use explicit-reasoning and hypothesis-testing processes to achieve this performance advantage. Their solutions are verbal, and conscious, and reportable to others. Humans instantiate a particularly powerful and agile form of category-rule learning. There may be brain circuits well organized for the rule- and hypothesis-testing processes of RB category learning. But then which species have them? It may also be that language helps humans hold rule-based hypotheses and apply them. But then what kinds of rule processes are possible without language? From either perspective, the evolutionary emergence of rule-based category learning is intriguing and deserving of study.

## 11. Rule-Based Categorization—Comparative Results

### 11.1. Pigeons

**Figure 14:**
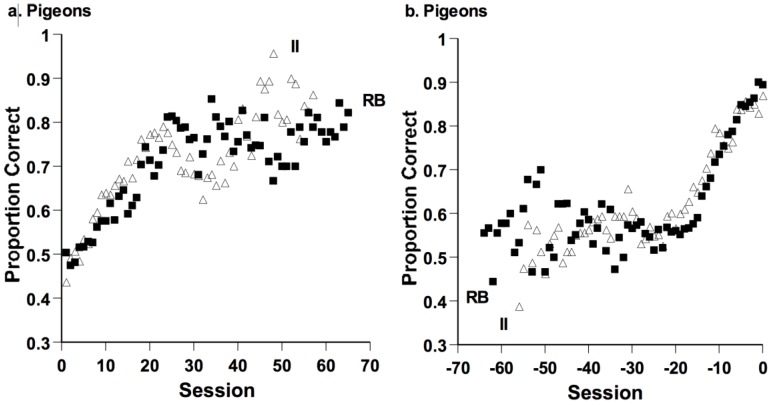
Pigeons performing information-integration (II) and rule-based (RB) tasks. (**a**) Proportion of correct responses in each session from the onset of learning forward for eight II-learning pigeons (open-triangle symbols) and eight RB-learning pigeons (filled-square symbols); (**b**) proportion of correct responses in each session from the criterial block backward for eight II-learning pigeons and eight RB-learning pigeons. Reprinted from [58]. Copyright 2012 by Elsevier. Reprinted with permission.

Smith *et al.* evaluated pigeons’ RB-II performance in a normal operant-discrimination situation [59]. The stimuli were circular sine-wave gratings varying in bar spatial frequency (width) and orientation (tilt). Figure 14a shows performance by session from the onset of learning for eight RB and eight II learning pigeons. These forward learning curves (learning graphed forward from the beginning of acquisition) show no hint of any RB performance advantage. However, pigeons were removed from the task upon reaching the criterion, leaving the weaker performing birds to be graphed subsequently. This adds waviness to the acquisition curves. Accordingly, Figure 14b shows performance by sessions backward from the criterion block. In these backward learning curves, the birds’ approach to criterion is aligned, but still there is no evidence of an RB advantage. Pigeons showed no difference in the speeds of learning or the final performance levels in the two tasks. To the contrary, these graphs seem to reflect that pigeons were learning two category tasks of the same character and difficulty. Pigeons showed no preference for one-dimensional task solutions or for tasks affording something like rule formation.

This result points to a notable weakness in pigeons’ selective-attention capacity. It is as though within the context of this task they could not direct attentional resources selectively toward one dimension. This surprising result complements that of Pearce *et al.* [60], who also demonstrated a striking failure of selective attention by pigeons, leading them to question whether pigeons even possess central attention-allocation processes.

This result also suggests that the psychological privilege accorded dimensional analysis and one-dimensional category rules is not a vertebrate-wide cognitive adaptation. Pigeons’ category learning might illuminate a phylogenetically ancient associative categorization system that is still predominant in some vertebrate lines. We could be seeing the character of the stem vertebrate category-learning systems before dimensional analysis and category rules evolutionarily augmented it.

Pigeons’ indifference to the task’s rotation in stimulus space is like humans’ indifference to task rotations within non-separable stimulus spaces that defeat the analytic processes of selective attention [61,62]. Possibly during category learning in these tasks, pigeons treat stimuli as integral wholes, not applying attentional, dimensional, or rule selection/focus. Pigeons’ data patterns could reflect that they bring the same implicit category system to bear on both RB and II category tasks. That system would not be affected by whether the category task is dimensionally aligned or not, and learning would proceed equivalently either way. Pigeons’ performance is consistent with a cognitive organization by which they gradually associate behavioral responses to regions of perceptual space or to unanalyzed stimulus wholes, while withholding (or lacking the capacity for) stimulus analysis, selective attention, and rule formation. This interpretation recalls Pearce’s configural theory [12].

There might be advantages to having a unitary category-learning system based on responding to configural wholes. It would reduce strategy competition from multiple processes competing to solve tasks. One would need no referee or metacognitive agency to adjudicate this competition. One could avoid the superstitious rules that humans sometimes “discover” when their rule system prevails wrongly. A configural system might also be especially adept at learning complex or non-linear category decision boundaries that would defeat a dimensionally aligned, rule-based system. Moreover, many natural kinds might well be organized essentially as high-dimensional II tasks (recalling Rosch’s probabilistic feature theory) and thus well suited to holistic (configural) responding [32,63].

### 11.2. Macaques (Macaca Mulatta)

Smith *et al.* tested rhesus macaques (an Old-World non-human primate) on RB-II category tasks [56]. The macaques performed these tasks in counterbalanced order (RB-II; II-RB). Smith *et al.* found similar results in a companion experiment with capuchin monkeys (*Cebus apella*—a New World primate) [64]. These monkeys at the Language Research Center (LRC) had been trained to respond to computer-graphic stimuli by manipulating a joystick that controlled a cursor. The LRC pioneered the exploration of primates’ cognitive systems using computer-based stimulus presentation and joystick-controlled behavioral responses by the monkeys. This work originated and continues with the express goal that these cognitive-testing techniques can enrich animals’ lives cognitively by providing forms of cognitive stimulation like that humans deliberately seek out (as when your child seeks out Minecraft!).

Macaques’ capacity for explicit cognition is not a salient feature of their cognition. They have relatively smaller frontal cortices than humans [65], producing performance decrements on tasks that foster response competition or need response inhibition [66,67]. Therefore, one reasonable hypothesis was that they might have a minimal rule-based category-learning system [12]. In that case, RB and II tasks should be learned about equally well and fast, because the tasks are matched in category structure and inherent difficulty unless the organism can apply dimensional attention or rule-based categorization selectively to the RB task.

**Figure 15:**
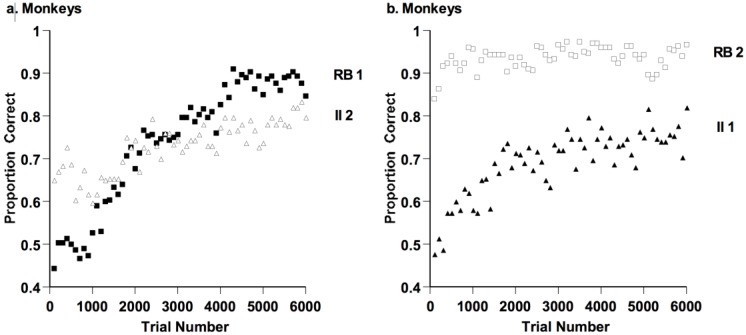
(**a**) Proportion of correct responses in each 100-trial block for three monkeys who performed 6000 trials of a rule-based (RB) and an information-integration (II) category task, in that order; and (**b**) proportion of correct responses in each 100-trial block for three monkeys who performed 6000 trials of an II and an RB category task, in that order. Reprinted from [58]. Copyright 2012 by Elsevier. Reprinted with permission.

Macaques’ proportion correct by block (100 trials per block) is shown in Figure 15. Macaques learned the RB task faster and to a higher final performance level than the II task. In the RB-II task order (Figure 15a), macaques improved from a very low RB performance level (as they initially acclimated to the task and stimuli) to a very high level later on. In the II-RB task order (Figure 15b) they were sharp and sensitive RB categorizers nearly from the task’s outset, having earlier accomplished their acclimation. Strikingly, taking only the first 600 trials of their second task, they were 90% and 67% correct on the RB and II task, respectively. Dimensional analysis clearly had privilege within their category-learning system. Despite lesser frontal-cortical brain development and their lack of language, macaques have similarities to humans in their RB-II performance.

These monkey results suggest answers to several of the comparative questions raised earlier. It is not the case that language is a necessary condition for the psychological privilege toward rules or dimensional analysis to emerge. Early multiple-system theories tied explicit categorization and category rules to verbal hypotheses [19,27]. The monkey results cut this tie. Dimensional hypotheses or criteria for categorization can apparently be represented successfully in mind as non-verbal proto-rules or cortical loops of sustained dimensional-focusing activity.

The results also show that the privilege toward dimensional analysis in categorization is not a humanly unique thing. It extends beyond humans, at least to other species in the order *Primates*. This implies that rule-based categorization had an earlier evolutionary origin than with humans. It raises the additional questions of when that dimensionally analytic system of categorization emerged, why it was adaptive, and what the most primitive steps toward category rules may have been.

## 12. Category Rules and Representational Portability

Zakrzewski and her colleagues have begun research to explore these issues (e.g., [68]). She asks whether RB and II category knowledge are qualitatively different in cognitive content, and whether they are different in the same way for humans and macaques. If so, this would strengthen the homology between humans’ and monkeys’ RB category learning. In particular, we have tested the idea that the generalization of II and RB category knowledge should differ substantially—for humans and primates—for theoretical reasons described now.

Correct responding in II tasks is believed to be governed by immediate reinforcement that links correct responses to training stimuli. Therefore, II category learning should be yoked to those original training stimuli, and it should generalize to new stimuli with constraints determined by interstimulus distances and dissimilarities. Moreover, II learning is implicit. So there will be no freestanding category knowledge resident in working memory to build a bridge to correct generalization for novel stimuli. For this reason, too, II category knowledge should have limited generalizability to novel stimuli.

However, correct responding in RB tasks—for humans—is thought to depend on an organizing rule resident in working memory (this answer for the non-human primates is not known but it should be discoverable). This rule could be more abstract than II category learning is. It could have substantial stimulus independence. A color rule, for instance, might survive changes in other perceptual features (shape, size, *etc.*). According to this theoretical account, RB category learning should be more generalizable. Casale *et al.* demonstrated this generalizability [69]. We took a step farther to demonstrate this generalizability in humans and macaques.

**Figure 16:**
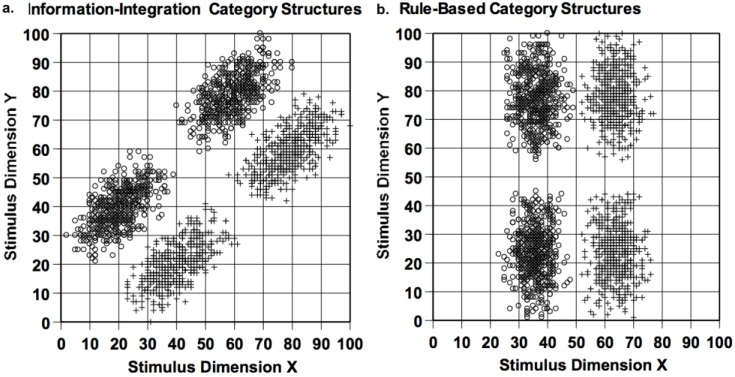
(**a**) A training-generalization information-integration category structure, depicted in the same way as Figure 12. The bottom pair and top pair of stimulus ellipses, respectively, were the defined categories during training and generalization; and (**b**) a training-generalization rule-based category structure, depicted in the same way.

Figure 16 shows our experimental situation [68]. We broke the category structures generally used in RB-II research (Figure 12) into training and transfer distributions. In this way, we could train with the category stimuli in the lower part of the stimulus space, and then suddenly test generalization by presenting stimuli from the untrained part of the space. In this experiment, we maintained trial-by-trial reinforcement after each trial, so that we are measuring transfer with ongoing learning.

The monkeys transferred RB learning seamlessly. They stayed at very high performance levels through the transition, so they generalized with no difficulty. This is consistent with the idea that they were applying some form of category knowledge that was more abstract with more stimulus independence, something beyond an associative response to the original training stimuli. In contrast, their II learning faltered, requiring additional trials to recover. This is consistent with some form of category knowledge that is less independent of training stimuli and less prepared to generalize. Humans’ result was strikingly similar, with flawless RB transfer but faltering II transfer. In this one respect, it does seem that RB learning is similarly distinctive for humans and macaques, in the sense that it was immediately portable to new and untrained stimuli.

However, remember that in this case, though RB knowledge immediately generalized, it did so in the context of always being propped up by immediate trial-by-trial reinforcement. Accordingly, we are asking now what happens in RB and II generalization if we knock away the props.

This experiment is conducted in three phases. First, we train participants on the Category A and B stimulus distributions in one part of the perceptual space. After they reached criterion, we gradually wean them away from trial-by-trial reinforcement, so that now they were performing blocks of trials with no reinforcement and receiving their summary feedback at the end of each trial block. This is a technique called deferred-rearranged reinforcement that we borrowed from earlier animal research (e.g., [70]), and human research [49]. Participants completed trial blocks with no feedback. After each block, they received together the reinforcements from all correct trials and then together the timeouts from all error trials. Through this technique, we were able to present familiar stimuli on most trials, sustaining existing category knowledge and making the task reinforcing enough so that everybody stayed on task. Under these conditions, though, we could also salt in transfer trials—from the untrained region of the category space—with no announcement or separation from the ongoing task, so that existing category knowledge would just transfer spontaneously—if it could. In this way, we also avoided the shock of suddenly moving participants to a testing phase with all novel stimuli. We thought this complementary approach was worthwhile for letting us evaluate whether the category knowledge that derives from original RB and II training is spontaneously, inherently different in generalizability.

The answer for humans is clear-cut [71]. In RB tasks, humans under deferred-rearranged feedback are completely able to transfer their rule knowledge to the occasional novel, untrained stimuli that are presented. Their II category knowledge, whatever form it exists, does not show this feature of portability at all. Perhaps this is because the category knowledge—the category learning—is welded associatively to the training stimuli. In the II case there is no verbal or conscious or symbolic representational material freestanding that can be applied to new stimuli.

Now Zakrzewski and her colleagues are testing macaques in this paradigm [72]. Preliminary results show that monkeys’ II learning—just as humans’ II learning—is unable to transfer to untrained stimuli while under deferred reinforcement. However, the crucial test is whether monkeys can transfer RB knowledge. Preliminary results suggest that this knowledge is substantially less portable than humans’ RB knowledge is.

Their transfer weakness bears on the extent to which primates’ rule-based performances fully involve something like category rules. Non-human primates may share some aspects of humans’ rule-based categorization, but not all. This result is potentially important to multiple-systems theory for suggesting that there are primitive and partial forms of rule use in categorization.

There is a lot to uncover, now, in order to understand why monkeys’ failure in RB transfer occurred. Perhaps it is because of a smaller working memory capacity. Perhaps it is because of a lack of language. Some of these reasons may be uncovered by work in developmental research in this area of RB category learning. Monkeys’ provisional failure and humans’ definite success in transferring RB knowledge *while under deferred reinforcement* show how distinctive humans’ ability really is. Humans clearly have a distinctive explicit system for rule-based categorization that monkeys are only on the way towards.

## 13. General Discussion

We described three theoretical perspectives on categorization (Section 2). From these arose the two theoretical debates that have dominated our literature: the debate on exemplars *vs.* prototypes (Section 3, Section 4, Section 5, Section 6 and Section 7) and the debate on single category-learning systems *vs.* multiple systems (Section 8, Section 9, Section 10, Section 11 and Section 12). These theories and debates provide a good grounding in the categorization literature. Then, we explored the contributions of recent animal-cognition research to this literature to reach these conclusions.

(1) Consider the exemplar-prototype debate first. Animals are poor at encoding specific exemplars that deserve particular responses. The disability is so serious it could be fatal to animals encountering exemplar-forcing natural categories. Therefore, it is unlikely that exemplar processing is the unitary system through which animals categorize. Assuming evolutionary continuity, the same may be true for humans. In fact, humans show converging performance failures when exemplar processing is forced on them (e.g., [5,73,74]). Murphy discussed these failures pointedly, reminding researchers to carefully attend to them and to seek to understand ecologically-valid categorization [42,43]. Doing so can give insight into when and why exemplars or prototypes are used in categorization.
(2) Animals commonly pre-assume that categories will be organized by family resemblance and, possibly, prototype-based. Observing this pre-assumption, one must consider why it is appropriate or adaptive in the natural world. Animal studies encourage us to reach that understanding (Section 6 and Section 7) [8].
(3) Given animals’ exemplar weakness and prototype strength, one is led to ask whether animals’ systems for categorization reflect the structure of natural categories. Natural categories were the proving ground for categorization and could have provided a shaping evolutionary force. Anderson [75], Briscoe and Feldman [2], Feldman [76], Shepard [45,46], and Smith [8] have pointed to the possibility of these shaping or tuning forces, but the perspective of animal cognition brings them back to life. If family-resemblance categories were common in nature [32,48,77,78,79], then cognitive evolution would sensibly have drifted toward the family-resemblance pre-assumption. But if the ecology were different and exemplar-forcing, the drift of cognitive evolution might have been different, and animals might have achieved a different tradeoff between exemplar-specific and abstractive-general classifications.
(4) Thus, in this area, animals have made a very important theoretical statement about categorization. The hope that there can be a pan-exemplar description of categorization is essentially gone. In this conclusion, animal-cognition studies endorse converging findings from many cognitive, neuroscience, and neuropsychological studies showing that exemplar processing is insufficient to fully describe human categorization [1,21]. Animal-cognition research even reaches out to help refine our human theories. We stress that by this conclusion we are not rejecting exemplar theory, or exemplar processes, as having a role in animals’ and humans’ overall categorization system. We are rejecting, based on everything about categorization’s natural history, that there can be a unitary, comprehensive exemplar theory.
(5) Animals have also made important contributions to the debate about single and multiple categorization systems. Pigeons’ equivalent performance in RB and II tasks is a reminder that some organisms, sometimes, may perceive the world integrally (in Garner’s sense) [55], not separably, and holistically, not analytically. If so, then RB and II tasks will be identical in learning and performance as pigeons show. One does not have to selectively attend. One does not have to analyze and narrow in on one dimension. One can just make the correct response to whole stimuli after those responses have been associatively strengthened through reinforcement. Attention is optional in a significant, unappreciated sense. Moreover, pigeons suggest that in the stem vertebrate categorization system selective attention may have played a lesser role, though we would not deny pigeons’ some form of selective attention in some situations.
(6) In fact, it is known that pigeons can, over multiple training sessions, gradually change the focus of something like attention in the service of improving performance within operant discriminations (e.g., [80]). This shows that the idea of selective attention is a nuanced construct, for pigeons gradually shift the axis of their discriminative learning over sessions, but they seem not to recruit dimensional attention in an RB task so as to gain a performance advantage there. Monkeys, in contrast, do recruit some attentional or rule process that grants them a meaningful RB advantage. Why?
(7) Zakrzewski’s RB-II research shows that the notion of a category rule is also a nuanced construct that deserves more theoretical attention. Rules for humans bring many cognitive affordances, including the important advantage of making category knowledge freestanding and easily portable to novel, untrained stimuli. Our preliminary results show that monkeys share that affordance only partially, and there may be other affordances of rules that they do not share. The full theoretical description of category rules needs to include an understanding of the degrees of separation between monkeys’ rule-like processes and humans’ rule-based processes, and consider the possible evolutionary pathways that may connect them.
(8) In the end, comparative RB-II research carries the headline of evolutionary emergence and discontinuity, from pigeons’ integral categorization to humans showing robust, explicit, declarative, language-based rule systems. Even here, animals illuminate the theoretical issues of human categorization by showing how profound the cognitive changes have been in the evolution of category rules.
(9) Clearly, the hope for a pan-single system category theory is lost to the human literature as well. Humans do have a profoundly sophisticated rule system for category learning, one that is apparently served by different brain processes and neural circuits, and one very different from that expressed in other vertebrate lines. It is time for our whole field to fully respect the contributions of cognitive neuroscientists who have demonstrated this system fully and elegantly.
(10) Perhaps it was inevitable that categorization—an essential backbone of animal cognition—would turn out to be important enough to have multiple cognitive systems supporting it, so that even the same organism can bring different processes to bear on different category tasks. We may capture the richness of our field, and the richness of this capacity, more fully if we acknowledge this possibility.
(11) Animal-cognition research has also cast light on the different high-level choices that theorists can make approaching a cognitive capacity. They may try to enforce a structural parsimony and unitarianism onto the science from the top down, or they can hold back and accept the psychological processes and systems they glean when research occurs from the bottom up. In the area of comparative categorization, as in the area of the cognitive neuroscience of categorization, the latter approach has proven more successful. Minds just may not be parsimonious, no matter our wishes in the matter.

Thus, animal-cognition research has made important contributions to our understanding of human and animal categorization, by prompting the field to give categorization an evolutionary depth, and phylogenetic breadth. In this way we have been led to consider the state of nature, the survival needs of animals, evolutionary tuning forces, adaptive approaches, deep continuities across vertebrate lines, but also strong emergence across them. We believe that the perspective from evolutionary depth could also illuminate other areas of human cognition. And we believe that animal-cognition research will continue to provide a synergistic complement to human research in future years.
